# Supplementary material for: Dynalign II: common secondary structure prediction for RNA homologs with domain insertions
Source: Nucleic Acids Res. 2014 Nov 21;42(22):13939–48. doi: 10.1093/nar/gku1172 (PMC4267632; doi:10.1093/nar/gku1172)
Supplement: SUPPLEMENTARY DATA [file supp_gku1172_nar-02021-z-2014-File012.zip › manual/Text/RemovePseudoknots.html]

RNAstructure Command Line Help: RemovePseudoknots


|  |  |  |
| --- | --- | --- |
|  | RNAstructure Command Line Help RemovePseudoknots | - Contents - Index |
| RemovePseudoknots takes a structure (or structures) and breaks pairs to generate a pseudoknot-free structure (or structures) with either lowest folding free energy change or with fewest removed pairs. USAGE: RemovePseudoknots <input ct file> <output ct file> [options] Required parameters: <input ct file> \| The name of a CT file possibly containing structure(s) with pseudoknotted pairs.; <output ct file> \| The name of a pseudoknot-free CT file to which output will be written. Options which don't require added values: -d, -D, --DNA \| Specify that the sequence is DNA, and DNA parameters are to be used. Default is to use RNA parameters. (This has no effect if the -m, -M, --maximize option is chosen.); -h, -H, --help \| Display the usage details message.; -m, -M, --maximize \| Specify that remaining base pairs should be maximized in the pseudoknot-free structure. Default is that the structure(s) should have minimum free energy. Minimizing free energy change can result in fewer pairs in the pseudoknot free structure(s) than the maximum. Options which require added values: -t, -T, --temperature \| Specify the temperature at which calculation takes place in Kelvin. Default is 310.15 K, which is 37 degrees C. References: 1. Reuter, J.S. and Mathews, D.H. "RNAstructure: software for RNA secondary structure prediction and analysis." *BMC Bioinformatics*, 11:129. (2010). 2. Smit, S., Rother, K., Heringa, J. and Knight, R. "From knotted to nested RNA structures: a variety of computational methods for pseudoknot removal." *RNA*, 14:410-416. (2008). | | |
| Visit The Mathews Lab RNAstructure Page for updates and latest information. | | |
